# An Essential Role for Katanin p80 and Microtubule Severing in Male Gamete Production

**DOI:** 10.1371/journal.pgen.1002698

**Published:** 2012-05-24

**Authors:** Liza O'Donnell, Danielle Rhodes, Stephanie J. Smith, D. Jo Merriner, Brett J. Clark, Claire Borg, Belinda Whittle, Anne E. O'Connor, Lee B. Smith, Francis J. McNally, David M. de Kretser, Chris C. Goodnow, Chris J. Ormandy, Duangporn Jamsai, Moira K. O'Bryan

**Affiliations:** 1 Prince Henry's Institute of Medical Research, Clayton, Victoria, Australia; 2 The Department of Anatomy and Developmental Biology, Monash University, Melbourne, Victoria, Australia; 3 Australian Phenomics Facility, John Curtin School of Medical Research, Australian National University, Canberra, Australian Capital Territory, Australia; 4 Medical Research Council Centre for Reproductive Health, The Queen's Medical Research Institute, University of Edinburgh, Edinburgh, United Kingdom; 5 Department of Molecular and Cellular Biology, University of California Davis, Davis, California, United States of America; 6 The Garvan Institute of Medical Research, Sydney, New South Wales, Australia; The Jackson Laboratory, United States of America

## Abstract

Katanin is an evolutionarily conserved microtubule-severing complex implicated in multiple aspects of microtubule dynamics. Katanin consists of a p60 severing enzyme and a p80 regulatory subunit. The p80 subunit is thought to regulate complex targeting and severing activity, but its precise role remains elusive. In lower-order species, the katanin complex has been shown to modulate mitotic and female meiotic spindle dynamics and flagella development. The *in vivo* function of katanin p80 in mammals is unknown. Here we show that katanin p80 is essential for male fertility. Specifically, through an analysis of a mouse loss-of-function allele (the Taily line), we demonstrate that katanin p80, most likely in association with p60, has an essential role in male meiotic spindle assembly and dissolution and the removal of midbody microtubules and, thus, cytokinesis. Katanin p80 also controls the formation, function, and dissolution of a microtubule structure intimately involved in defining sperm head shaping and sperm tail formation, the manchette, and plays a role in the formation of axoneme microtubules. Perturbed katanin p80 function, as evidenced in the Taily mouse, results in male sterility characterized by decreased sperm production, sperm with abnormal head shape, and a virtual absence of progressive motility. Collectively these data demonstrate that katanin p80 serves an essential and evolutionarily conserved role in several aspects of male germ cell development.

## Author Summary

Microtubules are critical components of cells, acting as a “scaffold” for the movement of organelles and proteins within the cytoplasm. The control of microtubule length, number, and movement is essential for many cellular processes, including division, architecture, and migration. We have defined the role of the microtubule severing protein katanin p80 in male germ cell development. Male mice carrying a point mutation in the p80 gene are sterile as a consequence of low numbers of sperm, abnormal sperm morphology, and poor motility (ability to “swim”). We show that this mutation is associated with defects in microtubule structures involved in the division of immature sperm cells, in structures that shape the sperm head, and in the sperm tail, which is essential for sperm movement in the female reproductive tract. This study is the first to show that katanin p80, via its effects on microtubule dynamics within the testis, is required for male fertility.

## Introduction

The regulation of microtubule dynamics is an essential requirement for all cells and in many aspects of their daily function. The ability to precisely regulate microtubule number, the assembly of networks, and the rate of microtubule assembly and disassembly underpins cellular processes including division, differentiation and migration. Male gamete development in particular relies upon the co-ordinated development and rapid remodelling of complex microtubule structures, such as the mitotic (spermatogonia) and meiotic (spermatocyte) spindle; flagella formation needed for sperm motility; and the manchette, which determines sperm head shape and contributes to tail structure. Approximately one in 20 men of reproductive age is sub-fertile or sterile, of which 60% of cases are due to intrinsic defects in spermatogenesis. This heterogeneous disorder manifests clinically as diminished sperm number, or abnormal motility or morphology, or commonly combinations thereof, in the ejaculate [1]. All of these clinical presentations may be underpinned by defective microtubule dynamics.

Microtubule severing is emerging as a key regulator of microtubule dynamics [2], [3], [4], [5], . The most well characterized microtubule severing enzyme is the katanin complex [8], the severing function of which is carried out by an ATPase enzymatic subunit, named p60, encoded by the *Katna1* gene. Katanin p60 is a member of the AAA domain (ATPases Associated with diverse cellular Activities) protein family. Upon binding ATP, katanin p60 oligomerizes onto the tail of an individual tubulin subunit within a microtubule to form a 14–16 nm ring structure [9]. ATP hydrolysis confers a conformational change in the oligomer and ‘tugs’ upon the tail of the tubulin subunit. This leads to destabilization, and ultimately severing, of the polymer [2]. Other AAA microtubule-severing proteins include spastin and fidgetin [8]. Mutations in the gene encoding spastin cause progressive axon degeneration and underlie ∼40% of autosomal dominant cases of hereditary spastic paraplegia [10] and deletion of the fidgetin gene in mice results in a severe behavioural and developmental phenotype [11], illustrating the importance of the family in neuronal development.

The regulation and compartmentalization of microtubule severing is essential for normal cell function and survival. Katanin p60-mediated severing can be modulated by a p80 regulatory subunit, encoded by the *Katnb1* gene [9]. The p80 subunit of katanin binds to p60 and targets it to the centrosome in transfected mammalian cell lines [9], [12], and generally enhances severing, but can also inhibit it depending on the cellular context [4], [12]. In *Tetrahymena thermophila* [13] and *Caenorhabditis elegans* [14], p80 null mutants phenocopy p60 null mutants. However the *in vivo* role of the p80 subunit in mammals remains enigmatic. Katanin was identified as a heterodimer of p60 and p80 subunits in sea urchins [8], however the ratio of the two subunits shows developmental and regional variation in rat neurons [15] and in mouse testis (the current study), suggesting that the expression of the p80 regulatory subunit may be one way in which p60-mediated severing is controlled in mammals [4]. The *Katnb1* gene contains a C-terminal WD40 domain predicted to be involved in protein-protein interactions, and as such is a strong candidate for targeting p60-mediated severing to particular locations within a cell and for targeting p60-mediated severing to post-translationally modified or microtubule-associated protein (MAP)-associated tubulin polymers [16], [17]. Katanin p80 also binds to the molecular motor protein dynein [18] and to dynein-regulating proteins [18], [19] and could thus be involved in the transport of the katanin complex to specific sites.

Katanin function is evolutionarily conserved, with p60 and p80 orthologues identified in species from all 5 kingdoms, including in *C. elegans, Drosophila melanogaster, Arabidopsis thaliana, Chlamydomonas reinhardtii*, mice and humans. Katanin localizes to mitotic spindle poles in mammalian cell lines, where it regulates spindle structure and chromosome movement [2], [3], [20], [21]. Mutations in *C. elegans* orthologues of katanin p60 and p80 reveal roles for katanin in oocyte meiotic spindle assembly and chromosome movement [20], [22], and katanin regulates different microtubule populations, including kinetochore-associated bundles, to control oocyte meiotic spindle length in *Xenopus* [23]. Of note, katanin regulates mitotic chromosome movement in *D. melanogaster* by participating in the so-called “Pacman-mediated” shortening of spindle microtubule plus-ends which results in the poleward movement of the chromosomes [24]. In addition, mutations in katanin orthologues in two distantly related organisms *Tetrahymena* and *Chlamydomonas* result in the absence of the central axoneme microtubule pair and cilia/flagella defects, indicating a role in ciliogenesis [13], [25], [26]. To date there have been no *in vivo* models of katanin dysfunction in mammals.

Here we show that a missense mutation in the highly conserved WD40 domain of the *Katnb1* gene, encoding the p80 regulatory subunit of katanin, causes male sterility in mice characterized by oligoasthenoteratozoospermia. *Katnb1* mutant mice, denoted as Taily, show frequent failure of meiotic spindle resolution, defective manchette function and abnormal axoneme development. Our findings highlight the critical role for katanin p80 in the regulation of microtubules dynamics in many aspects of male germ cell development. These data raise the possibility that defective katanin function may also contribute to human infertility, specifically defective sperm number, morphology and/or sperm motility.

## Results

### Infertility in Taily mice is caused by a point mutation in the *Katnb1* gene

**Figure 1:**
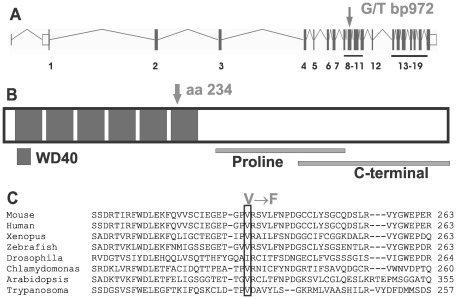
The Taily mutation within the *Katnb1* gene and protein. A. The position of the mutation in the WD40 region of the *Katnb1* gene (arrow) and protein (B). The Taily mutation (arrow) results in the conversion of a valine (GTC) to a phenylalanine (TTC) in the WD40 repeat region of the katanin p80 protein. C. A cross-phyla comparison of the region of the *Katnb1* gene containing the Taily mutation.

**Figure 2:**
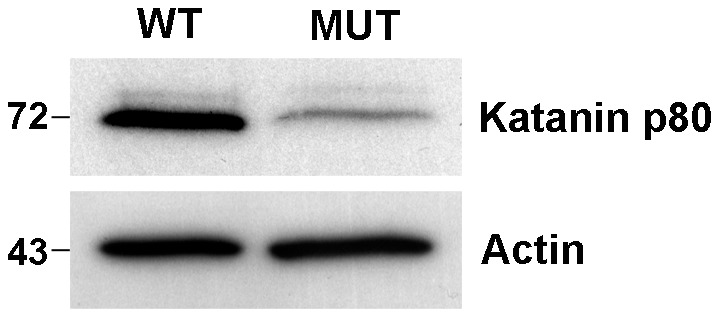
Reduction in katanin p80 protein in haploid germ cells from *Katnb1^Taily/Taily^* mice. Round spermatids were isolated from *Katnb1^WT/WT^* (WT) and *Katnb1^Taily/Taily^* (MUT) mice. 10 µg of protein from each cell preparation was immunoblotted for katanin p80, and actin as a protein loading control. Molecular weights are shown on the left. Of note, the molecular weight of the p80 subunit in mice is 72 kDa as predicted by Ensembl (www.ensembl.org).

Mouse lines carrying ENU-induced mutations causing male sterility were identified using breeding trials as described previously [27]. Lines with G3 male sterility at a frequency of one in four, but with normal mating behaviour, were chosen for further analysis. They included the ‘Taily’ line. The causal mutation was mapped using SNP-based methods and ultimately narrowed to a linkage interval on chromosome 8 (between SNP markers rs3089148 and rs3710112) containing 74 genes. Candidate genes were chosen on the basis of testis expression and proposed function. The protein-coding region and intron-exon boundaries of 30 genes were sequenced. The causative mutation in Taily mice was identified as a recessive G to T substitution in exon 9 of the *Katnb1* gene. No other mutations were found. Unaffected males possessed either homozygous wild type alleles (*Katnb1^WT/WT^*) or were heterozygous for the wild type and Taily allele (*Katnb1^WT/Taily^*). Greater than 50 mice of each genotype were assessed and the genotype-phenotype correlation was absolute. The Taily mutation resulted in the conversion of a valine (GTC) to a phenylalanine (TTC) in the WD40 repeat region of the katanin p80 protein (Figure 1). The presence of an aliphatic amino acid (e.g. V or I) at amino 234, relative to the mouse sequence, is absolutely conserved across all species, and is strongly suggestive of a functionally important role (Figure 1B). Western blot analysis revealed that haploid germ cells from *Katnb1^Taily/Taily^* mice contained markedly less p80 protein than those from *Katnb1^WT/WT^* mice (Figure 2), demonstrating that the ‘Taily’ allele likely results in a loss-of-function.

### The katanin p80 regulatory subunit is essential for male fertility

*Katnb1^Taily/Taily^* males showed no overt behavioural abnormalities, were morphologically identical to wild type littermates, were of normal weight (Figure S1), but were uniformly sterile when mated with wild type females (n≥10, *Katnb1^Taily/Taily^* males aged ≥8 weeks of age). *Katnb1^Taily/Taily^* females had apparently normal fertility.

**Figure 3:**
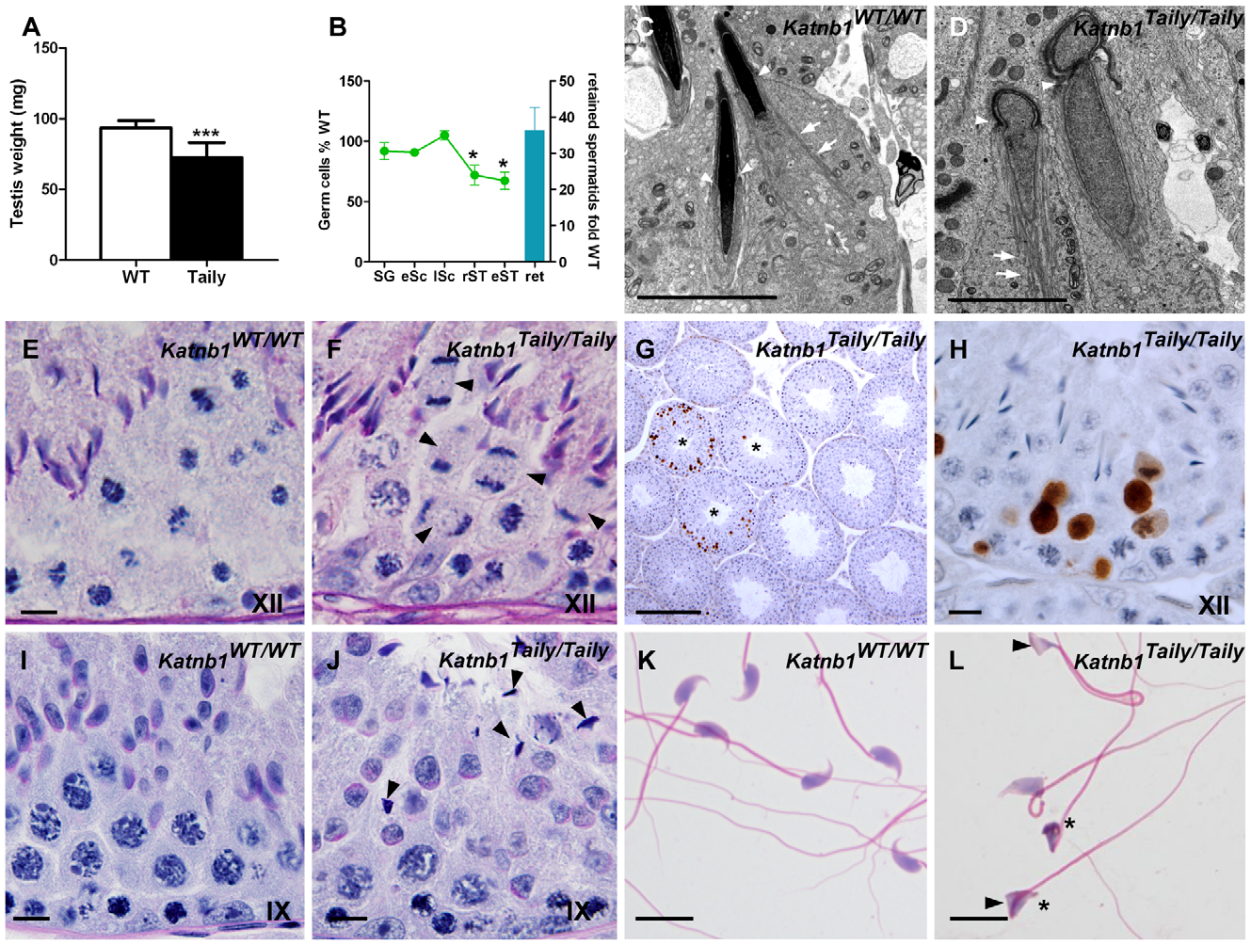
Spermatogenic defects in Taily mice. A. Testis weight in wild type (WT) (n = 14) and *Katnb1^Taily/Taily^* mice (n = 22). B. Stereological analysis of germ cell numbers per testis in *Katnb1^Taily/Taily^* versus WT mice (n = 5–6). Numbers of germ cells per Sertoli cell were expressed as %WT (left hand axis). The number of spermatids retained within Sertoli cells after spermiation was expressed as fold WT (right hand axis). Abbreviations: SG, spermatogonia; eSc, early spermatocytes; lSc, late spermatocytes; rST, round spermatids; eST, elongating and elongated spermatids; ret, retained spermatids. In A, B, data are mean ± SD, * denotes p<0.05, *** denotes p<0.001 *Katnb1^Taily/Taily^* versus WT. C, D. Electron microscopy of sperm nuclear shape in elongating spermatids from WT (C) and *Katnb1^Taily/Taily^* (D) mice. White arrowheads indicate the perinuclear ring, which shows an abnormal constriction in spermatids from *Katnb1^Taily/Taily^* mice (D). White arrows indicate the manchette microtubules. Scale bar in C,D = 5 µm. E, F. Chromosome segregation in WT (E) and *Katnb1^Taily/Taily^* (F) mice. Arrow heads indicate cells stalled in anaphase of meiosis I. Scale bar in E, F = 10 µm. G, H. TUNEL analysis of apoptotic cells in *Katnb1^Taily/Taily^* mice. Apoptotic cells were predominantly seen in stage XII and I tubules (asterix in G). Scale bar in G = 200 µm, H = 10 µm. I, J. Spermiation in WT (I) and *Katnb1^Taily/Taily^* (J) mice. Retained elongated spermatids (arrowheads) were frequently observed being phagocytosed by Sertoli cells in stage IX tubules from *Katnb1^Taily/Taily^* (J) but were rarely observed in WT (I) mice. Bar in I, J = 10 µm. K, L. Cauda epididymal sperm morphology from WT (K) and *Katnb1^Taily/Taily^* (L) mice. Taily sperm frequently displayed a hammer-head shape (arrowheads). Sperm heads were often bent back towards the tail (asterisk). Scale bar in K, L = 10 µm. Roman numerals indicate the stage of the spermatogenic cycle.

Testes from adult (8–12 weeks) *Katnb1^Taily/Taily^* mice were 18.7% smaller than those from wild type littermates (p<0.0001, Figure 3A). Seminiferous tubules contained all germ cells types. Two major discordant features were apparent within the seminiferous epithelium from *Katnb1^Taily/Taily^* mice: 1) abnormally shaped spermatid heads (Figure 3C and 3D) and 2) abnormal meiotic cells at metaphase-anaphase (Figure 3E and 3F). Stereological analysis revealed that the number of Sertoli cells, spermatogonia and spermatocytes per testis was not different between genotypes (Figure 3B and Table S1), indicating that the initiation of spermatogenesis and entry into meiosis were unaffected by the Taily mutation. The latter finding suggested that the function of the Sertoli cell blood-testis-barrier was normal, a proposition supported by the appearance of normal inter-Sertoli cell junctions by electron microscopy (not shown). By contrast, testes from *Katnb1^Taily/Taily^* mice contained ∼30% fewer spermatids (round and elongating) compared to wild type (Figure 3B). The reduction in spermatid populations was due to a decrease in the number of cells exiting meiosis, specifically during the final meiotic division in stage XII (Figure 3B and Table S1). TUNEL-labelling revealed that apoptotic cells were predominantly present in stage XII and stage I tubules which is when the final events of meiosis occur (Figure 3G). Apoptotic spermatocytes in the process of meiotic division were observed (Figure 3H). Collectively these results indicate that katanin p80 function is required for the final phases of male meiotic cell division.

**Figure 4:**
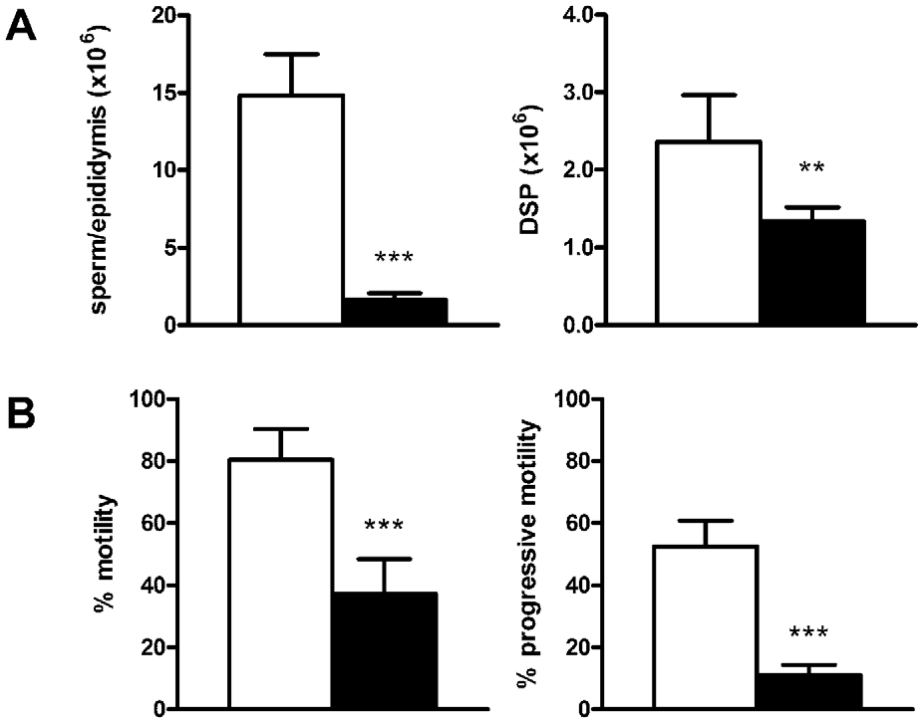
Sperm output and functional characteristics. A. Total epididymal sperm content (n = 7/group) (left hand panel) and daily sperm output (DSP, right hand panel) by the testes (n = 6/group) from wild type (white bars) and *Katnb1^Taily/Taily^* (black bars) mice. B. The percentage of sperm showing any form of motility (left hand panel) and those showing progressive motility (right hand panel) in wild type (white bars) and *Katnb1^Taily/Taily^* (black bars) mice (n = 6–7/group). Data are mean ± SD, ** denotes p<0.01, *** p<0.001 compared to WT.

Stereology also showed that additional germ cells were not lost as they progressed through spermiogenesis (Table S1), however there was a 36 fold increase in the number of spermatozoa being phagocytosed by Sertoli cells (Figure 3B) in stages IX–XI tubules (Figure 3I and 3J). These data indicate a failure in spermiation, the process by which sperm are released by the Sertoli cell at the end of their development, prior to their passage to the epididymis [28]. The data show that a significant proportion of spermatozoa failed to be released from the Sertoli cell and were instead phagocytosed, thus leading to a reduced number of sperm entering the epididymis. As a consequence, the epididymides from *Katnb1^Taily/Taily^* males contained a lower total number of sperm than would be anticipated from the testicular daily sperm output, i.e. 11% of wild type in the epididymis compared to 57% in the testis (Figure 4A). Of the sperm found in the cauda epididymis of Taily mice, when compared to wildtype (Figure 3K), all had abnormally shaped heads (Figure 3L) and displayed compromised total motility as assessed by computer assisted sperm analysis (80.3% in *Katnb1^WT/WT^* versus 37.2% in *Katnb1^Taily/Taily^*) (Figure 4B). Very few sperm were capable of forward (progressive) motility (52.4% in *Katnb1^WT/WT^* versus 11.1% in *Katnb1^Taily/Tail^*) (Figure 4B).

Collectively these data indicate that katanin p80 has a role in germ cell exit from meiosis, in the establishment of structures or pathways within the sperm tail involved in motility and in the shaping of the sperm head. *Katnb1^Taily/Taily^* males were sterile as a consequence of decreased sperm production, abnormal sperm morphology and sperm being unable to ascend the female reproductive tract following mating. The analogous human phenotype is referred to as oligoasthenoteratozoospermia (low sperm count, poor motility and abnormal shape).

### Expression of katanin p80 and p60 subunits in the mouse testis

**Figure 5:**
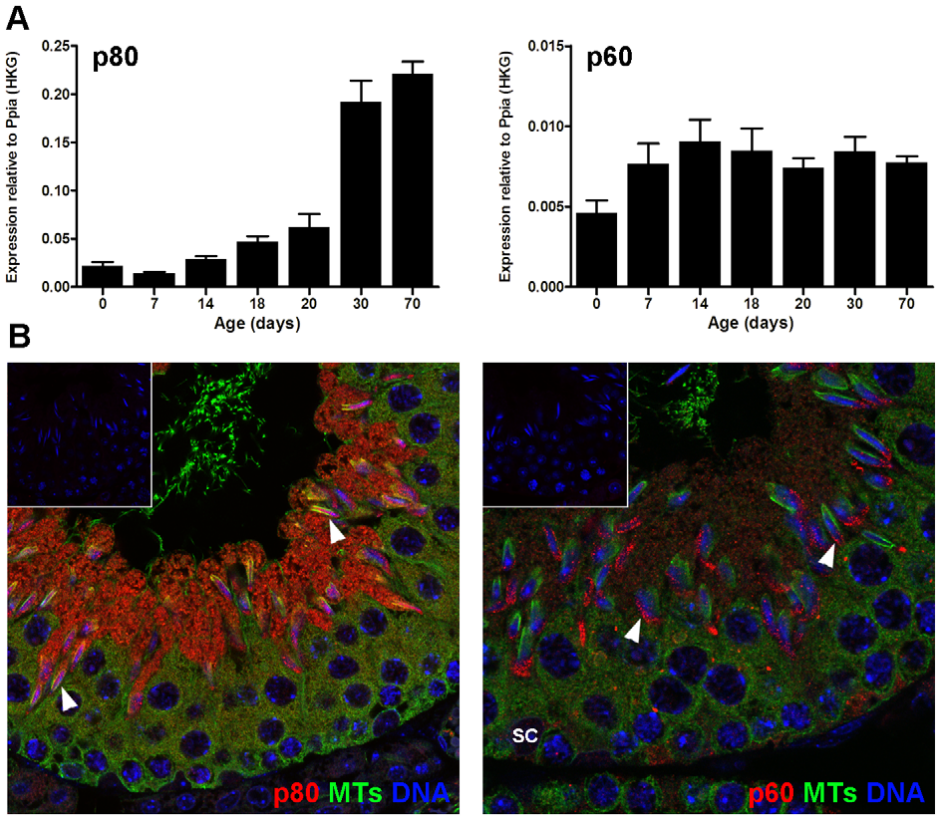
Katanin p60 and p80 expression in the mouse testis. A. Quantitative RT-PCR expression data for katanin p80 (left hand panel) and p60 (right hand panel) during post-natal testis development. Expression data was normalized against *Ppia* (housekeeper gene expression) and shown as mean ± SEM, n = 3. B. Immunohistochemical localization of katanin p60 (right) and p80 (left) in the mouse seminiferous epithelium. Katanin subunits = red, α-tubulin = green, nuclei = blue. Both katanin subunits were prominently localized to elongating spermatids (arrowheads). Katanin p60 was also visible in Sertoli cell nuclei (SC). The inset in each micrograph shows a control for the secondary antibody (primary antibody substituted with buffer).

Both katanin p80 and p60 mRNAs were expressed in the testis during the post-natal establishment of the spermatogenic cycle (Figure 5). The katanin p60 microtubule severing enzyme was expressed at relatively similar levels in all ages examined, suggesting expression in multiple cell types (Figure 5A). Katanin p80 regulatory subunit expression, however, peaked at day 30, suggesting predominant expression in post-meiotic haploid spermatids (Figure 5A). These data are consistent with previous microarray data (germonline.org) indicating that the katanin p60 catalytic subunit is expressed in Sertoli cells and germ cells to a similar degree, whereas the p80 regulatory subunit while detectable in Sertoli cells and spermatogonia, is more highly expressed (5-fold) in spermatocytes and spermatids [29]. In accordance with the mRNA data, katanin p80 protein was most strongly localized within round through to elongating spermatids (Figure 5B). Katanin p60 was also prominent in spermatids and was visible in Sertoli cells (Figure 5B).

In addition, the katanin p60 orthologues *Katnal1* (p60-like 1) and *Katnal2* (p60-like 2) were also expressed within the developing post-natal testis with a timing similar to that observed for katanin p80 (Figure S2). Absence of KATNAL1 immunolocalization in germ cells (Smith et al, submitted for publication) and immunolocalization of KATNAL2 predominantly to the sperm tail and cytoplasm, but not associated with the sperm head, (Figure S2), suggests that the meiotic and spermatid head-shape phenotypes reported herein were primarily mediated by katanin p80 regulation of the eponymous p60 subunit.

### Katanin p80 regulates microtubule dynamics during male meiotic division

**Figure 6:**
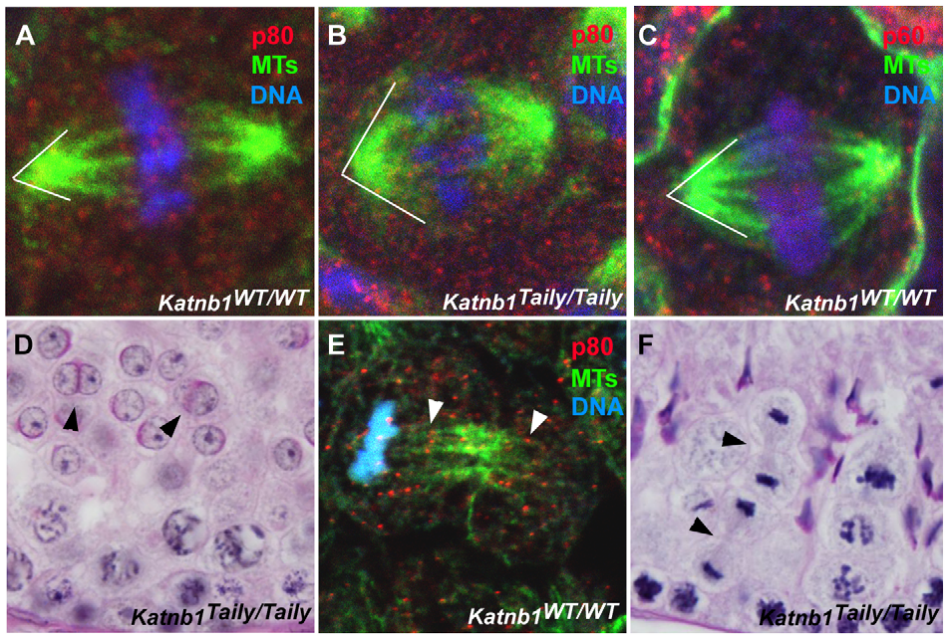
Defective meiotic division in *Katnb1^Taily/Taily^* male gametes. A, B. Katanin p80 localization and meiotic spindle structure in wild type (WT) (A) and *Katnb1^Taily/Taily^* (B) testes. White lines indicate the angle at which microtubules project from spindle poles. C. Katanin p60 localization to meiotic spindles in WT mice. D. Binucleated haploid spermatids (arrowheads) in *Katnb1^Taily/Taily^* testes. E. Katanin p80 localization to mid-bodies in meiotic telophase. Arrowheads indicate p80 localization at midbody microtubules. F. Late telophase cells with prominent midbody structures (arrowheads) were observed in *Katnb1^Taily/Taily^* but not WT testes. In A, B, C and E red represents katanin subunits, green represents microtubules (MTs) as labelled by α-tubulin immunostaining, and blue represents DNA as labelled by DAPI.

The loss of germ cells during meiotic division prompted an investigation of the microtubule-based meiotic spindle in *Katnb1^Taily/Taily^* males. Compared to *Katnb1^WT/WT^* littermates, all *Katnb1^Taily/Taily^* meiotic spindles were abnormal (Figure 6A and 6B). Specifically, metaphase spindles appeared to be more densely populated with microtubules, and projected from the poles at a wider angle than those from wild type animals (Figure 6A and 6B and Videos S1 and S2). Pole-to-pole measurements in metaphase spindles were longer in *Katnb1^Taily/Taily^* compared to *Katnb1^WT/WT^* (12.15±0.16, n = 58, versus 10.52±0.17 µm, n = 78, mean ± SEM, p<0.0001).

Within metaphase and anaphase cells, p60 (Figure 6C and Videos S1 and S2) and p80 (Figure 6A and 6B) proteins localized to microtubules of meiotic spindles. The *Katnb1^Taily/Taily^* mutation did not overtly alter this localization. Both p60 and p80 were observed along the microtubules of the spindle and at the microtubule-chromosome interface. The latter localization is consistent with katanin involvement in the poleward movement of chromosomes in *Drosophila* mitotic cells [24]. Specifically within *Drosophila*, katanin is believed to participate in the depolymerization of microtubule plus-ends in the midzone at anaphase during *Drosophila* mitosis, effectively “chewing away” the microtubule ends to facilitate spindle shortening via a process known as “Pacman” [24]. A role for katanin p80 in the Pacman-mediated poleward movement of chromosomes in mammalian meiotic anaphase is further supported by the appearance of multiple cells stalled in late anaphase in *Katnb1^Taily/Taily^* mice (Figure 3F).

Disordered meiosis is further evidenced by the frequent occurrence of binucleated haploid spermatids in *Katnb1^Taily/Taily^* testis sections (Figure 6D). Binucleated spermatids were never observed in wild type animals. These data strongly suggest a role for katanin p80 in cytokinesis and midbody resolution. This hypothesis is supported by the localization of katanin p80 (Figure 6E) and p60 (Figure S3) to the microtubules of the midbody in late telophase cells in both *Katnb1^WT/WT^* (Figure 6E) and *Katnb1^Taily/Taily^* germ cells (Figures S3 and data not shown). In addition, telophase cells with prominent midbody microtubules were observed in testes from *Katnb1^Taily/Taily^*, but not in wild type animals (Figure 6F). Taken together, the results suggest that katanin p80, most likely in association with p60, has a prominent role in midbody dissolution in male meiotic cells, and that this function is disrupted in Taily mice.

### Katanin p80 regulates microtubule-based structures essential for normal sperm morphology and motility

Abnormal sperm head shape (Figure 3D and 3L) is frequently associated with defects in the function of the manchette [30], [31], [32]. The manchette is a transient microtubule structure assembled in elongating spermatids with proposed roles in both the sculpting of the sperm head and in the movement of proteins destined for the sperm tail, via a process referred to as intra-manchette transport (IMT) [33]. The manchette is comprised of large, parallel arrays of microtubule bundles that extend from beneath the acrosome/acroplaxome region of the spermatid head and project into the spermatid cytoplasmic lobe containing the growing sperm tail ([34] and Figure S4). The manchette is first seen at step 8 of spermiogenesis, when the round spermatid nucleus polarizes to one side of the cytoplasm, and the spermatid commences elongation. Nucleation of microtubules in the manchette is thought to occur on the perinuclear ring region of the spermatid head (Figure S4), and large parallel bundles are assembled as the spermatid nucleus starts to change shape in step 9.

In order to investigate the hypothesis that head abnormalities in sperm from *Katnb1^Taily/Taily^* mice were the consequence of abnormal manchette structure or function, testis sections were examined using electron microscopy. Manchettes in wild type elongating spermatids displayed the characteristic perinuclear ring and microtubule array structure (Figure 3C). Those from *Katnb1^Taily/Taily^* elongating spermatids, however, displayed several abnormalities including constricted perinuclear rings, nuclear distortion and abnormally long microtubules extending into the distal cytoplasm (Figure 3D).

**Figure 7:**
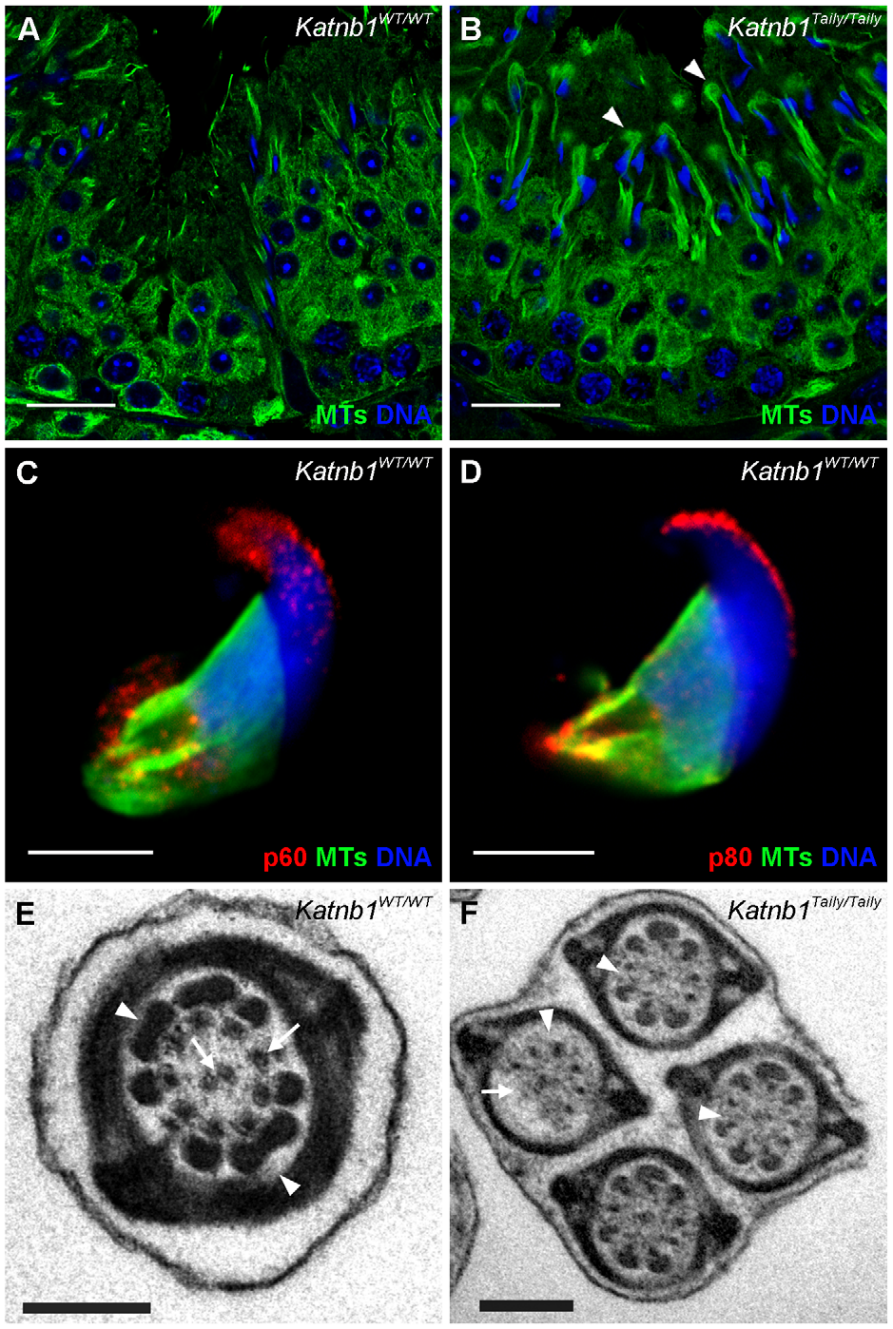
Katanin localization to manchettes and abnormalities in manchette resolution and flagella structure in *Katnb1^Taily/Taily^* spermatids. A. Immunolocalization of α-tubulin (microtubules, MTs) in stage I, during which time manchette resolution occurs in wild type mice. B. The localization of α-tubulin in a stage I tubule from a *Katnb1^Taily/Taily^* mouse. Arrowheads indicate abnormal “clouds” of tubulin in the spermatid cytoplasm at the time of manchette disassembly. In A and B, green = microtubules (MTs), blue = DNA (DAPI), scale bar in B = 20 µm. C, D. Immunolocalization of katanin p60 (C) and katanin p80 (D) in wild type spermatids. Scale bar = 5 µm. Red = katanin subunits, green = α-tubulin (microtubules, MTs), blue = DNA (TOPRO). E, F. Structure of sperm flagella in *Katnb1^WT/WT^* (E) and *Katnb1^Taily/Taily^* (F) sperm. Scale bar = 0.5 µm. In E, arrowheads indicate normal outer dense fiber structures and arrows indicate microtubules of the axoneme. In F, arrowheads indicate abnormal or missing outer dense fibers, and arrows indicate missing axonemal microtubules.

A stage-by-stage comparison of sections from wild type and *Katnb1^Taily/Taily^* males suggested defective manchette resolution in mutant animals. Although manchettes eventually resolved, the removal of manchettes in *Katnb1^Taily/Taily^* males was delayed. In wild type mice, manchettes normally reduce in size and then disappear in step 13 spermatids. In contrast, and when compared with wild type mice (Figure 7A), in step 13 spermatids from *Katnb1^Taily/Taily^* mice, abnormally long manchette microtubules extended into the cytoplasm and were associated with tubulin-labelled ‘clouds’ (Figure 7B). The timing and location of the ‘clouds’ is suggestive of abnormal microtubule disassembly.

**Figure 8:**
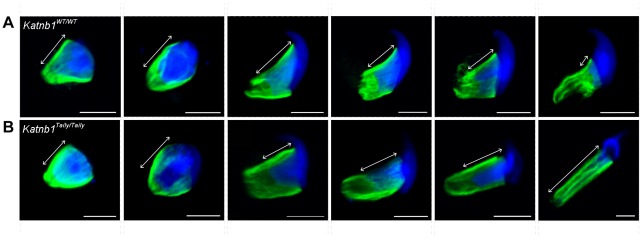
Manchette structure in wild-type and *Katnb1^Taily/Taily^* spermatids. A. α-Tubulin staining (green) of manchette microtubules in isolated spermatids from WT mice. Elongating spermatids are shown in progressive steps of manchette development from left to right. Arrows indicate the distance between perinuclear ring of the manchette and the posterior portion of the nucleus. B. α-Tubulin staining (green) of manchette microtubules in isolated spermatids from *Katnb1^Taily/Taily^* mice. Arrows indicate the distance of the peri-nuclear ring to the posterior portion of the nucleus. In all images, blue staining = DNA (TOPRO). The scale bar in each micrograph = 5 µm.

The above defects were confirmed and more dynamically visualized in elongating spermatids isolated from wild type and *Katnb1^Taily/Taily^* males labelled with α-tubulin and TOPRO to visualize microtubules and the nucleus, respectively (Figure 8 and Videos S3 and S4). An analysis of progressively more mature elongating spermatids revealed that while manchettes in *Katnb1^Taily/Taily^* mice appeared to form at the correct time and initially began to move distally as spermiogenesis proceeded, movement stalled at approximately step 10 (Figure 8). By contrast, the progressive constriction of the peri-nuclear ring that normally occurs as the manchette moves over the caudal half of the spermatid, continued to occur (Figure 8). This resulted in a bulbous nuclear shape forward of the stalled peri-nuclear ring, and an abnormally elongated nucleus distally, resulting in the unusual ‘knob-like’ head structure also visible at an electron microscopic level (Figure 3D). As observed by electron microscopy (Figure 3D) and in testis sections (Figure 7B) abnormal elongated manchette microtubules were easily observed in isolated late step spermatids (Figure 8).

Consistent with the defects seen in *Katnb1^Taily/Taily^* mice, both the katanin p80 regulatory subunit and the p60 catalytic subunit localized to microtubules of the manchette (Figure 7C and 7D). Focal labelling of katanin subunits was observed along the manchette microtubules and particularly at the microtubule ends projecting into the cytoplasm (Figure 7C and 7D). This localization is consistent with a role for katanin-mediated severing in regulating manchette length. Localization was not obviously affected in the manchette of *Katnb1^Taily/Taily^* mice (not shown). Both subunits also localized to the acrosome/acroplaxome region, in a manner reminiscent of proteins that undergo trafficking in an acrosome/acroplaxome-manchette-flagella pathway [35].

Collectively these results reveal that katanin p80, most likely in association with p60, has an essential role in both the formation of the manchette and in the dynamics of its movement and resolution. Perturbed katanin p80 function results in the failure of manchette migration, abnormally long and mal-orientated manchette microtubules and abnormal dissolution. Such abnormalities are entirely consistent with the observed defects in sperm head shape (teratozoospermia) in sperm from *Katnb1^Taily/Taily^* animals.

The manchette also plays a critical role in the development of sperm flagella via a process known as intra-manchette transport, or IMT [33]. IMT is thought to be involved in sperm tail development in a manner analogous to intra-flagella transport in somatic cilia and in flagella in organisms including *Chlamydomas* and Trypanosomatids [36]. Defects in manchettes and sperm motility in *Katnb1^Taily/Taily^* mice, and a previously demonstrated role for katanin p80-mediated (PF15p) severing in the formation of axoneme central microtubules in *Chlamydomonas* [26], prompted us to investigate flagella/tail structure in *Katnb1^WT/WT^* and *Katnb1^Taily/Taily^* sperm. When compared to controls (Figure 7E), electron microscopic analysis revealed a variety of axoneme defects, including missing central microtubules and hemi-axonemes (Figure 7F). Of note, the majority of *Katnb1^Taily/Taily^* sperm also contained flimsy or missing outer dense fibers (Figure 7F) consistent with previously proposed roles for the manchette in the transport of proteins into the developing sperm tail and formation of accessory tail structures [33]. The outer dense fibers are rod-like structures running parallel to, and connected to, the microtubules of the axoneme that are believed to protect sperm against shearing forces and to provide directionality to tail bending (reviewed in [37]). Collectively, these defects demonstrate an essential role for katanin p80 in the development of the sperm flagellum, including the axoneme and the formation of the accessory structures.

## Discussion

Analysis of male mice with a mutation in the regulatory subunit of the katanin microtubule severing enzyme complex revealed several roles for katanin p80 in mammalian microtubule dynamics. These studies reveal an essential requirement for katanin p80 in male fertility, and in multiple aspects of mammalian male gamete development, including in meiotic spindle dynamics, cytokinesis, flagella development and sperm head shaping.

Together with *in vitro* data and evidence of disordered microtubule structure and function in lower order species, this novel mouse mutation reveals that katanin has roles in controlling microtubule dynamics. While severing can result in microtubule destruction, it is also important for the creation of new microtubules, via the severing of longer stable microtubules into shorter segments that can then be used as “seeds” for further microtubule polymerization [4], [7]. For example, katanin severs newly created microtubules at the neuronal centrosome, facilitating their transport to other sites, such as within developing axons [38]. Katanin can sever microtubule lattice defects in a quality control mechanism [39], and in neurons can create branched microtubule networks [4], [40]. Recent data also revealed that katanin can depolymerize microtubules at their plus-ends [5], [16], [39] and finally, the p60-p80 katanin complex has a severing-independent, microtubule cross-linking function at *C.elegans* oocyte meiotic spindle poles [23].

The p80 protein contains a WD40 domain that likely mediates protein-protein interactions [9], [12]. The C-terminal region interacts with the p60 enzyme [12] and contains binding sites for the molecular motor protein dynein. The C-terminal region also binds to the dynein-associated proteins LIS1 and NDE1 in neurons [18]. While p80 is thought to modulate p60 targeting and activity [3], [9], [12], the precise *in vivo* roles of p80 are not well understood. The *Katnb1^Taily/Taily^* mutation in the WD40 domain results in decreased p80 protein within germ cells and defects in microtubule-based processes. Based on the position of the mutation, and on the fact that less p80 protein is produced in mutant mice, we predict that the mutation influences the ability of p60 to sever, as well as the targeting of this severing activity to specific sites within the cell. This mouse model recapitulates many of the proposed functions of katanin observed in lower order species. It is of note, that the manchette defects observed in Taily mice phenocopy many of the defects observed in a *Lis1* null mice [31]. This observation and previous studies showing the localization of LIS1 and NDE1 in the manchette [41], suggests that similar to the proposed role for these proteins in neurons, the katanin complex co-operates with LIS1 and NDE1 during sperm head shaping. This interaction will be the subject of future investigations.

The data demonstrate a role for katanin p80 in mammalian male meiotic cell division. Null mutations in the *C.elegans* p80 ortholog *mei2* are associated with meiotic defects in oocytes, including an inability to assemble a meiotic spindle [14]. Katanin function in male germ cells has not previously been studied to the best of our knowledge. Our observations on male metaphase spindles in *Katnb1^Taily/Taily^* mice are consistent with the longer metaphase meiotic spindles produced in *C.elegans* oocytes with a partial loss-of-function *mei2* mutation [20] and with the recent demonstration of a conserved role for katanin in controlling the length of meiotic metaphase spindles in *Xenopus* oocytes [23].

Within *C.elegans*, the p80 protein targets the p60 severing enzyme to the spindle poles in meiotic oocytes [20], [42]. We did not observe either p60 or p80 at spindle poles in male meiotic germ cells. All meiotic spindles were, however, abnormal, indicating a role for katanin in the assembly of spindles, as supported by various studies [14], [20], [21], [23]. In meiotic male germ cells, the most obvious localization of katanin subunits was to the microtubule ends near the chromosomes in metaphase-anaphase cells, suggesting a role for katanin in the shortening of microtubule plus ends during anaphase. The “Pacman-mediated” shortening of microtubule plus-ends within the spindle midzone is important for the poleward movement of chromosomes in mitotic anaphase [24], but has not been studied in meiotic cells. Our observation of cells apparently stalled in anaphase, together with the finding that 30% of cells die during the later phases of meiosis, supports the hypothesis that disturbed p80 function causes defects in the poleward movement of chromosomes during anaphase. Such defects result in disturbed spindle resolution and, often, cell death. Finally, the appearance of binucleated spermatids and the localization of katanin subunits to the midbody in meiotic cells in male mice supports the hypothesis that p80, and potentially the katanin complex, has a conserved role in modulating microtubule dynamics at the midbody during meiotic cytokinesis. In support, katanin p80 dysfunction or mislocalization is associated with defective mitotic cytokinesis in *Trypanosomes* [25] and in sarcoma cells *in vitro* [43].

Katanin p80 is essential for sperm head shaping via the regulation of the manchette, which is in itself a complex microtubule network. An analysis of manchette position during spermiogenesis indicated that manchette movement is defective in *Katnb1^Taily/Taily^* mice, suggesting katanin is involved in the organization and remodelling of this microtubule network as it moves over the nucleus. The localization of p60 and p80 within the manchette, together with the Taily phenotype is consistent with a role for katanin action at multiple sites. These include 1) the severing of microtubules at the perinuclear ring, thereby facilitating the release of microtubules from the nucleating center, and the production of the microtubule lattice, as has been proposed in other systems [4]; 2) the severing of microtubules near the nucleus to permit movement of the manchette perinuclear ring as it shapes the nucleus; 3) within the microtubule lattice to facilitate remodelling of this complex structure; and 4) the severing of microtubules at the caudal end of the manchette to control manchette length and dissolution. Katanin activity also regulates the dynamics of large microtubule-based array structures in neurons [18], [38], [40]. Taken together, the data support the hypothesis that p80, and katanin function, is important for the movement and remodelling of large microtubule arrays in mammalian cells. This role is essential for the normal development and shaping of sperm, which in turn is critical for normal sperm function and male fertility.

Finally, we demonstrate for the first time that katanin p80 is required for mammalian sperm flagella development and subsequent motility. A conserved role for katanin in the assembly and disassembly of cilia and flagella has been revealed in two distantly related lower order species *Chlamydomonas* [26] and *Tetrahymena* [13] (reviewed in [6]). Katanin activity controls flagellum length in Trypanosomatids [25] and severs axonemal microtubules during the deflagellation process in *Chlamyodmonas* [44], [45]. In *Chlamydomonas*, katanin p80 is also specifically required for the assembly of the central microtubule doublet of the flagellum axoneme [26]. Defects in axonemal structures (including missing central microtubule doublets) and outer-dense fibers in *Katnb1^Taily/Taily^* mice suggest that katanin p80 regulates sperm motility by acting at multiple sites in sperm development. Specifically, p80 function is required in the regulation of axonemal assembly and in the delivery of proteins to the developing flagellum via IMT.

Given the high level of expression of katanin p80 in other tissues, and the proven role for katanin in *C.elegans* oogenesis, it is surprising that other overt phenotypes were not noted in mutant mice. We hypothesize that other phenotypes will be revealed when mice are exposed to environmental insults. Studies into the role of katanin in oocyte function are ongoing.

In conclusion, the p80 subunit of the katanin microtubule severing enzyme complex is required for male fertility in mice. This is the first *in vivo* mammalian model of katanin p80 dysfunction, and it presents with a phenotype reminiscent of a commonly observed clinical phenotype of male infertility characterized by low sperm counts, poor motility and abnormal sperm morphology (referred to as oligoasthenoteratospermia or OAT). We conclude that p80 katanin is required for male meiotic spindle development and dynamics, and for the shaping of the sperm head via the regulation of manchette development and movement. Katanin p80 also participates in meiotic cytokinesis, likely via the regulation of the microtubules within the midbody, and controls the development and function of sperm flagella.

## Materials and Methods

### Ethics statement

All animal experimentation was approved by the Australian National University and Monash University Animal Experimentation Ethics Committees and performed in accordance with Australian NHMRC Guidelines on Ethics in Animal Experimentation.

### Identification of the Taily mouse line and the causal mutation

Point mutant mice were generated as described previously on a C57BL/6 background and outbred to CBA [27]. Mouse lines containing sterility causing mutations were identified by breeding trials wherein eight G3 brother-sister pairs per line were co-housed and the presence of pups monitored. Lines where male sterility was observed in a ratio of approximately one in four in the G3 generation with apparently normal mating behaviour were selected for further analysis.

Affymetrix 5K mouse SNP Chip arrays were used to map the sterility causing mutation. Genomic DNA from five affected males was hybridized onto the array at the Australian Genome Research Facility and compared to wild type C57BL/6 and CBA sequences. The linkage interval was subsequently narrowed using additional mice and SNPs (www.well.ox.ac.uk/mouse/INBREDS/) using the Amplifluor SNP Genotyping System (Chemicon). Plates were read in a BMG Fluostar optima fluorescent microplate reader.

Following the identification of the causal mutation, mice were specifically genotyped using the Amplifluor SNPs HT genotyping system using a wild type-specific antisense primer 5′-GAAGGTCGGAGTCAACGGATTAAGAGCACCCGTACCTGAC-3′, a mutant allele antisense primer 5′-GAAGGTGACCAAGTTCATGCTGAAGAGCACCCGTACCTGAA-3′, a sense primer, 5′-GGTGGTGAGCTGCATTGAA-3′ and Platinum *Taq* DNA Polymerase (Invitrogen). Conditions for amplification were as follows: 4 minute denaturation at 95°C, 35 cycles of denaturation at 95°C for 10 seconds, annealing at 60°C for 20 seconds and elongation at 72°C for 40 seconds, followed by a final 3 minute elongation at 72°C. Following the reaction, plates were read in a BMG Fluostar optima fluorescent plate reader.

### Infertility characterization

Infertility in the Taily mouse line was classified using the scheme outlined in Borg et al [46]. Daily sperm output and total epididymal sperm content were determined as described previously [47]. Sperm motility was assessed using computer assisted sperm analysis [48] and ultra-structure using electron microscopy [49]. Cauda epididymal sperm morphology was assessed following staining with hematoxylin. Cells undergoing apoptosis were visualized using the Apoptag kit (Millipore) as recommended by the manufacturer.

### Stereological analysis

The number of germ cells per Sertoli cell were enumerated in 25 µm thick, periodic acid Schiffs (PAS) stained methacrylate sections using the optical disector as previously described [50]. Retained elongated spermatids were counted in stage XI-XI [50] and expressed as fold wild type.

### Katanin subunit expression

RNA was extracted from testes at defined periods throughout post-natal development using TRIzol regents (Life Technologies), treated with DNase I (Ambion) and cDNA sythnesized using oligo-dT primers and SuperScript III reverse transcriptase (Life Technologies). The relative expression of *Katnb1*, *Katna1*, *Katnal1* and *Katnal2* were defined using quantitative PCR using TaqMan assays (Applied Biosystems) Mm01244795_m1, Mm00496172_m1, Mm00463780_m1 and Mm00510701_m1 respectively. Expression of these was normalized against peptidylprolyl isomerase A (Mm002342429_g1).

### Germ cell isolation

Germ cell sub-populations were purified using the Staput method as previously described [51]. Single cell suspensions were loaded onto a 2–4% continuous BSA gradient and elongated spermatids and round spermatids collected after a 3 hour and 3.5 hour sedimentation period, respectively. For immunofluorescent staining, gradient fractions were pelleted and resuspended in 4% paraformaldehyde (PFA) fixative for 2 hours on ice. Cells were then washed with PBS and spread onto slides.

### Western blotting

Protein was extracted from round spermatid fractions (>90% purity) using 20 µL M-PER buffer (Thermo Scientific). 10 µg of protein was separated on a 10% SDS-PAGE gel and probed for rabbit katanin p80 (HPA041165, which recognizes a C-terminal region of p80, Sigma Aldrich) and actin (A2066, Sigma Aldrich). Bound antibody was detected using a goat anti-rabbit IgG HRP (P0488, Dako) secondary antibody and an enhanced chemiluminescence (ECL Plus) detection kit (Amersham Biosciences).

### Immunofluorescent microscopy

Katanin subunits and α-tubulin were localized in testis sections as described [52]. Primary antibodies included: anti-α-tubulin (T5168, Sigma, diluted 1 in 5000), anti-katanin p60-like 2 (p60AL2, #sc-84855, Santa Cruz, diluted 1 in 100), anti-katanin p80 (diluted 1 in 200) [9] and anti-katanin p60 (diluted 1 in 200) [53]. Both p60 and p80 antibodies were affinity-purified from rabbits immunised against full length recombinant human proteins. These antibodies have been validated extensively and have been shown to recognize a single polypeptide in HeLa cells and a range of human tissues [9], [53]. Given the sequence homology between the p60 and p60L1 subunits, and between p80 and the uncharacterized c15orf29 subunit, there remains the possibility of partial cross-reactivity. Secondary antibodies included: Alexa Fluor 555 donkey anti-rabbit IgG (A-31572) and Alexa Fluor 488 donkey anti-mouse IgG (A-21202) (diluted 1 in 500). DNA was labelled using DAPI (Invitrogen). To define the localization of proteins within isolated elongating spermatids, cells were permeabilized in 0.2% Triton X-100 diluted in 10% normal horse serum (NHS) in PBS for one hour at room temperature. Non-specific labelling was minimized by blocking in 10% NHS in PBS for 30 minutes. Primary antibodies were diluted in 10% NHS in PBS and incubated overnight at 4°C. Secondary antibodies were diluted 1 in 200 and incubated at room temperature for 2 hours. DNA was labelled using TOPRO3 (Invitrogen, 1 in 200) or DAPI. Images were taken with an SP5 5-channel (Leica Microsystems) confocal microscope in the Monash University Microimaging facility. Metaphase spindle lengths were measured on α-tubulin and DAPI-stained sections from *Katnb1^WT/WT^* and *Katnb1^Taily/Taily^* mice using LAS AF (Leica Application Suite Advanced Fluorescence) software. Z-stacks of spindles and manchettes were collected at 0.5 µm intervals. Images were assembled using Adobe Photoshop. Test and subject images were adjusted uniformly across the image and between groups.

### Statistics

Differences between *Katnb1^WT/WT^* and *Katnb1^Taily/Taily^* mice were determined using unpaired t tests in GraphPad Prism 5.0.

## Supporting Information

Figure S1 Body weight (g) in *Katnb1^WT/WT^* (WT, n = 13) and *Katnb1^Taily/Taily^* (Mutant, n = 21) mice. No significant differences were observed.
(PDF)

Figure S2 Katanin orthologue expression in normal mouse testis. A. *Katnal1* and *Katnal2* mRNA expression during testis development. Expression data was corrected for *Ppia* (housekeeper gene expression) and shown as mean ± SEM, n = 3. B. Katnal2 immunolocalization (red) in adult mouse testis. Green = α-tubulin (microtubules, MTs), blue = DNA (DAPI).
(PDF)

Figure S3 Immunolocalization of katanin p60 (red) to microtubules of meiotic midbodies (arrows) in *Katnb1^WT/WT^* and *Katnb1^Taily/Taily^* mice. Green = α-tubulin (microtubules, MTs), blue = DNA (DAPI).
(PDF)

Figure S4 Diagram of the microtubule-based manchette in spermatids. The spermatid acrosome overlying the nucleus associates, via a specialized adhesion junction, with F-actin-containing “hoops” in the Sertoli cell cytoplasm. Beneath the acrosome is the acroplaxome and the closely associated perinuclear ring, from which the manchette microtubules are believed to emerge. The manchette microtubules extend into the cytoplasm, and are believed to progressively shape the spermatid head by exerting a force on the nucleus [30]. The manchette is also thought to participate in the delivery of proteins to the developing flagellum via a process known as intra-manchette transport [33]. Diagram adapted from [34].
(PDF)

Table S1 Stereological analysis of spermatogenesis in *Katnb1^WT/WT^* (WT) and *Katnb1^Taily/Taily^* (Taily) mice. ^a^ Data expressed as mean±SEM. * denotes p<0.05 compared to wildtype (WT) using unpaired t test. ^b^ Early spermatocytes include preleptotene to pachytene spermatocytes in stage VIII. ^c^ Late spermatocytes include pachytene and diplotene spermatocytes in stages IX–XI. ^d^ Conversion ratios were calculated by dividing the hourly production rates (HPR) of one cell population by the HPR of the preceding cell population in the sequence of spermatogenesis. Meiosis entry = preleptotene spermatocytes/type B spermatogonia; meiosis progression = pachytene+diplotene spermatocytes in stages IX–XI/preleptotene spermatocytes; meiosis exit = step 1–3 round spermatids/pachytene+diplotene spermatocytes in stages IX–XI; progression of spermiogenesis = steps 14–15 elongated spermatids/steps 1–3 round spermatids.
(DOC)

Video S1 3D imaging of p60 katanin in meiotic spindles in *Katnb1^WT/WT^* mice. Red = katanin p60, green = α-tubulin, blue = DNA (DAPI).
(AVI)

Video S2 3D imaging of p60 katanin in meiotic spindles in *Katnb1^Taily/Taily^* mice. Red = katanin p60, green = α-tubulin, blue = DNA (DAPI).
(AVI)

Video S3 3D imaging of spermatid manchettes in *Katnb1^WT/WT^* mice. Spermatids were isolated from *Katnb1^WT/WT^* mice and immunostained for microtubules to visualize the manchette. Green = α-tubulin, blue = DNA (TOPRO).
(AVI)

Video S4 3D imaging of spermatid manchettes in *Katnb1^Taily/Taily^* mice. Spermatids were isolated from *Katnb1^Taily/Taily^* mice and immunostained for microtubules to visualize the manchette. Green = α-tubulin, blue = DNA (TOPRO).
(AVI)
