# Supplementary figures and images for: An Essential Role for Katanin p80 and Microtubule Severing in Male Gamete Production
Source: PLoS Genet. 2012 May 24;8(5):e1002698. doi: 10.1371/journal.pgen.1002698 (PMC3359970; doi:10.1371/journal.pgen.1002698)

## Body weight

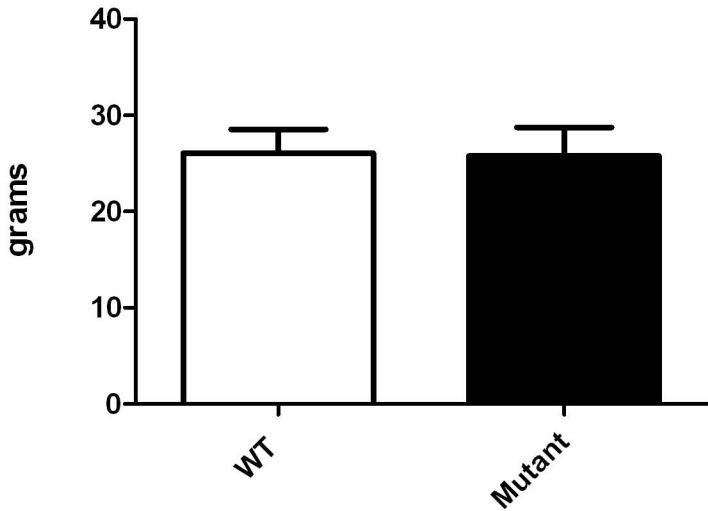

Supplement: Figure S1 — Body weight (g) in Katnb1WT/WT (WT, n = 13) and Katnb1Taily/Taily (Mutant, n = 21) mice. No significant differences were observed. (PDF) [file pgen.1002698.s001.pdf]

**A**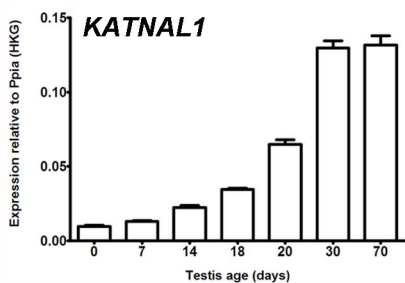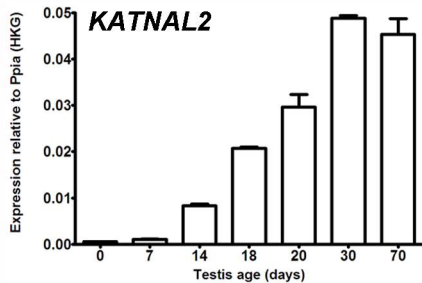**B**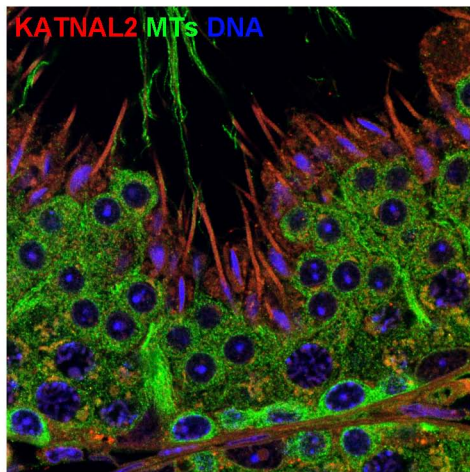

Supplement: Figure S2 — Katanin orthologue expression in normal mouse testis. A. Katnal1 and Katnal2 mRNA expression during testis development. Expression data was corrected for Ppia (housekeeper gene expression) and shown as mean ± SEM, n = 3. B. Katnal2 immunolocalization (red) in adult mouse testis. Green = α-tubulin (microtubules, MTs), blue = DNA (DAPI). (PDF) [file pgen.1002698.s002.pdf]

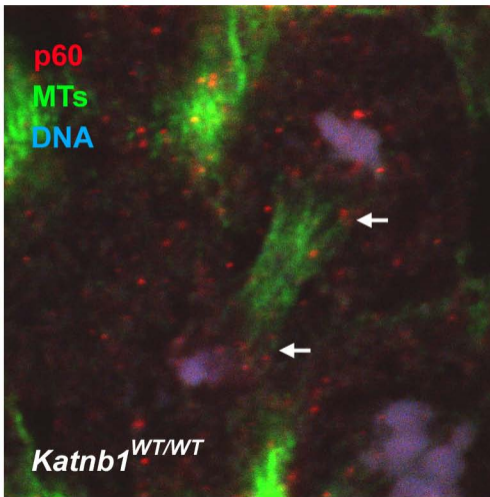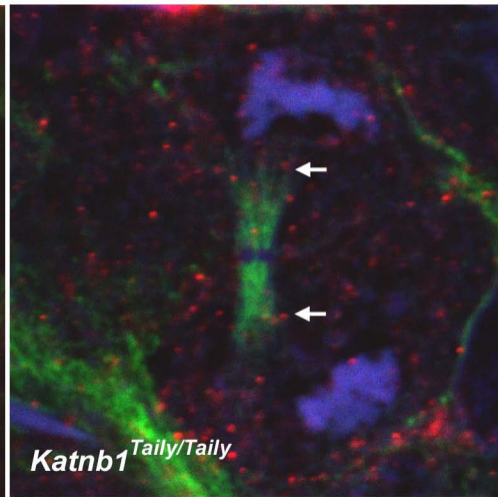

Supplement: Figure S3 — Immunolocalization of katanin p60 (red) to microtubules of meiotic midbodies (arrows) in Katnb1WT/WT and Katnb1Taily/Taily mice. Green = α-tubulin (microtubules, MTs), blue = DNA (DAPI). (PDF) [file pgen.1002698.s003.pdf]

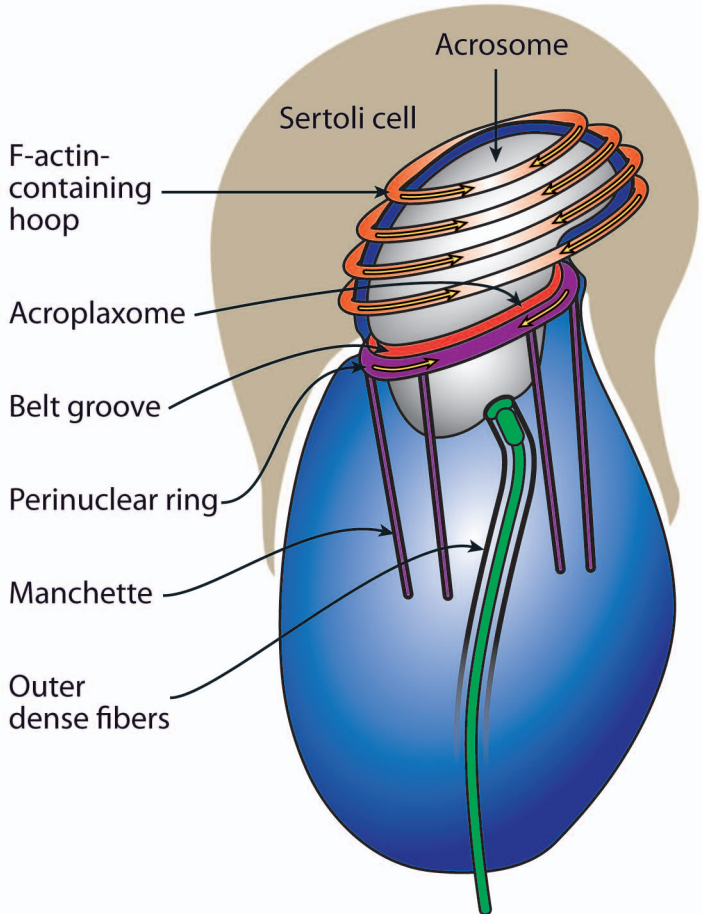

Supplement: Figure S4 — Diagram of the microtubule-based manchette in spermatids. The spermatid acrosome overlying the nucleus associates, via a specialized adhesion junction, with F-actin-containing “hoops” in the Sertoli cell cytoplasm. Beneath the acrosome is the acroplaxome and the closely associated perinuclear ring, from which the manchette microtubules are believed to emerge. The manchette microtubules extend into the cytoplasm, and are believed to progressively shape the spermatid head by exerting a force on the nucleus [30]. The manchette is also thought to participate in the delivery of proteins to the developing flagellum via a process known as intra-manchette transport [33]. Diagram adapted from [34]. (PDF) [file pgen.1002698.s004.pdf]
