# Supplementary material for: An Essential Role for Katanin p80 and Microtubule Severing in Male Gamete Production
Source: PLoS Genet. 2012 May 24;8(5):e1002698. doi: 10.1371/journal.pgen.1002698 (PMC3359970; doi:10.1371/journal.pgen.1002698)
Supplement: Table S1 — Stereological analysis of spermatogenesis in Katnb1WT/WT (WT) and Katnb1Taily/Taily (Taily) mice. a Data expressed as mean±SEM. * denotes p<0.05 compared to wildtype (WT) using unpaired t test. b Early spermatocytes include preleptotene to pachytene spermatocytes in stage VIII. c Late spermatocytes include pachytene and diplotene spermatocytes in stages IX–XI. d Conversion ratios were calculated by dividing the hourly production rates (HPR) of one cell population by the HPR of the preceding cell population in the sequence of spermatogenesis. Meiosis entry = preleptotene spermatocytes/type B spermatogonia; meiosis progression = pachytene+diplotene spermatocytes in stages IX–XI/preleptotene spermatocytes; meiosis exit = step 1–3 round spermatids/pachytene+diplotene spermatocytes in stages IX–XI; progression of spermiogenesis = steps 14–15 elongated spermatids/steps 1–3 round spermatids. (DOC) [file pgen.1002698.s005.doc]

**Supporting Table 1**. **Stereological analysis of spermatogenesis.**

|  | WT  n=4 | Taily ^a^  n=5 |
| --- | --- | --- |
| *Cell numbers (millions per testis)* |  |  |
| Sertoli cells | 2.81 ± 0.18 | 2.47 ± 0.21 |
| Type A spermatogonia | 1.54 ± 0.11 | 1.34 ± 0.11 |
| Type B spermatogonia | 1.52 ± 0.12 | 1.14 ± 0.20 |
| Early spermatocytes ^b^ | 16.7 ± 1.15 | 13.4 ± 1.10 |
| Late spermatocytes ^c^ | 2.72 ± 0.28 | 2.50 ± 0.24 |
| Round spermatids (steps 1-8) | 31.9 ± 3.45 | 19.8 ± 2.00* |
| Elongated spermatids (steps 9-15) | 39.3 ± 3.05 | 22.8 ± 2.31* |
| *Conversion ratios* ^d^ |  |  |
| Meiosis entry | 1.86 ± 0.50 | 1.76 ± 0.40 |
| Meiosis progression | 1.12 ± 0.20 | 1.48 ± 0.17 |
| Meiosis exit | 4.39 ± 0.26 | 2.81 ± 0.38* |
| Progression of spermiogenesis | 0.80 ± 0.07 | 0.84 ± 0.07 |

^a^ data expressed as mean±SEM. * denotes p<0.05 compared to wildtype (WT) using unpaired t test

^b^ Early spermatocytes include preleptotene to pachytene spermatocytes in stage VIII

^c^ Late spermatocytes include pachytene and diplotene spermatocytes in stages IX-XI

^d^ Conversion ratios were calculated by dividing the hourly production rates (HPR) of one cell population by the HPR of the preceding cell population in the sequence of spermatogenesis. Meiosis entry = preleptotene spermatocytes/type B spermatogonia; meiosis progression = pachytene + diplotene spermatocytes in stages IX-XI/preleptotene spermatocytes; meiosis exit = step 1-3 round spermatids/pachytene+diplotene spermatocytes in stages IX-XI; progression of spermiogenesis = steps 14-15 elongated spermatids/steps 1-3 round spermatids.
